# Epigenetic heterogeneity and plasticity in therapy‐induced tumor states through single‐cell multi‐omics

**DOI:** 10.1002/1878-0261.70285

**Published:** 2026-06-04

**Authors:** Hee Jung Kim, Hwiyeong Lee, Jin Hong, Daechan Park

**Affiliations:** ^1^ Ajou Energy Science Research Center Ajou University Suwon Korea; ^2^ Department of Molecular Science and Technology Ajou University Suwon Korea; ^3^ College of Advanced Bio‐Convergence Engineering Ajou University Suwon Korea

**Keywords:** cancer epigenetics, cell state plasticity, single‐cell multi‐omics, therapeutic resistance, tumor heterogeneity

## Abstract

Therapeutic resistance and disease recurrence remain major unresolved challenges in oncology, primarily driven by tumor heterogeneity and the inherent plasticity of cancer cells. Although multiple biological mechanisms contribute to these processes, epigenetic mechanisms are the key regulators of clonal diversification and adaptive transcriptional reprogramming under treatment pressure. This regulatory layer operates through reversible transcriptional changes that are independent of DNA sequence alterations, enabling cancer cells to respond to a selective environment. Recent advances in analytical methodologies, particularly single‐cell multi‐omics approaches, have markedly improved our capacity to dissect these regulatory processes at a single‐cell resolution. This review explores how diverse therapeutics, including chemotherapy, targeted agents, immunotherapy, hormonal interventions, and epigenetic drugs, induce the widespread remodeling of DNA methylation patterns, histone modifications, and chromatin accessibility. These therapy‐induced molecular changes drive transitions to distinct cellular states that confer survival advantages such as drug‐tolerant persister (DTP) phenotypes, senescence‐like populations, epithelial–mesenchymal transition (EMT) states, and immune‐evasive cell populations. We further evaluated the current single‐cell multi‐omics platforms for profiling chromatin‐based plasticity and identifying biomarkers with direct clinical relevance. Finally, we discuss how integrative multi‐layer analyses enable comprehensive characterization of tumor‐state evolution, providing a conceptual framework for precision oncology strategies aimed at overcoming resistance.

## Graphical abstract

Single‐cell multi‐omics reveals epigenetic heterogeneity across therapy‐adaptive tumor states, including quiescent/dormant, drug‐tolerant persister, and EMT‐like phenotypes. By linking regulatory features with state‐associated biomarkers, these approaches inform biomarker‐guided therapeutic strategies for evolving tumors.

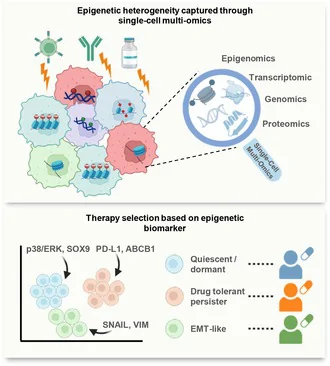

**Abbreviations**
AP‐1: activator protein‐1
AR: androgen receptor
CLL: chronic lymphocytic leukemia
DNMTs: DNA methyltransferases
DTP: drug‐tolerant persister
EGFR: Epidermal Growth Factor Receptor
EMT: epithelial–mesenchymal transition
ISR: integrated stress response
MHC: major histocompatibility complex
MOFA+: multi‐omics factor analysis plus
PD‐L1: programmed death‐ligand 1
PRC2: polycomb repressive complex 2
SASP: senescence‐associated secretory phenotype
scATAC‐seq: single‐cell assay for transposase‐accessible chromatin sequencing
scChIP‐seq: single‐cell chromatin immunoprecipitation sequencing
scCUT&Tag: single‐cell cleavage under targets and tagmentation
scMethyl‐seq: single‐cell methylation sequencing
scRNA‐seq: single‐cell RNA sequencing
SHARE‐seq: simultaneous high‐throughput ATAC and RNA expression sequencing
TET: ten‐eleven translocation
TF: transcription factor
TFs: transcription factors

## 1 Introduction

The survival rate of patients with metastatic solid tumors remains below 30% across most cancer types despite significant advances in cancer therapeutics over the past decades [1]. This ongoing clinical challenge largely arises from therapeutic resistance and metastasis, which account for most of cancer‐related deaths [2, 3]. Even therapies with high initial response rates ultimately fail owing to acquired resistance mechanisms that develop over time [4, 5]. Although genetic alterations contribute to drug resistance, mutation‐centric models are insufficient to explain the rapid and reversible drug tolerance observed shortly after treatment initiation. Moreover, drug‐tolerant persister (DTP) cells, which survive initial therapy without detectable resistance mutations, further highlight the role of nongenetic mechanisms [6, 7, 8]. Thus, adaptive resistance may arise from both genetic and nongenetic processes.

Epigenetic plasticity provides a mechanistic basis for nongenetic adaptive responses, enabling dynamic transitions between transcriptional states under therapeutic stress and generating heterogeneous subpopulations with differential drug sensitivities [6, 9]. However, conventional bulk approaches mask the cellular heterogeneity underlying these dynamic state transitions, limiting their ability to resolve these adaptive processes. Recent advances in single‐cell multi‐omics technologies have enabled the dissection of these processes by associating epigenetic states with transcriptional programs at single‐cell resolution [10, 11]. In this review, we examine how various anticancer treatments, including chemotherapy, targeted agents, immunotherapy, hormonal interventions, and epigenetic drugs, induce the widespread remodeling of DNA methylation patterns, histone modifications, and chromatin accessibility, thereby generating distinct cellular states that promote survival. We further discuss how single‐cell multi‐omics approaches can identify state‐associated biomarkers, uncover therapeutic vulnerabilities, and inform strategies for overcoming therapeutic resistance.

## 2 Therapy‐induced epigenetic remodeling and cell state evolution

Therapeutic resistance arises through dynamic tumor adaptation during treatment, a process shaped by evolutionary dynamics, and, in part, epigenetic regulation of cellular states [12]. In this section, we discuss how pretreatment epigenetic heterogeneity shapes the diversity of initial therapeutic responses and examine the epigenetic reprogramming induced by distinct treatment interventions [12, 13] (Fig. 1).

**Fig. 1:**
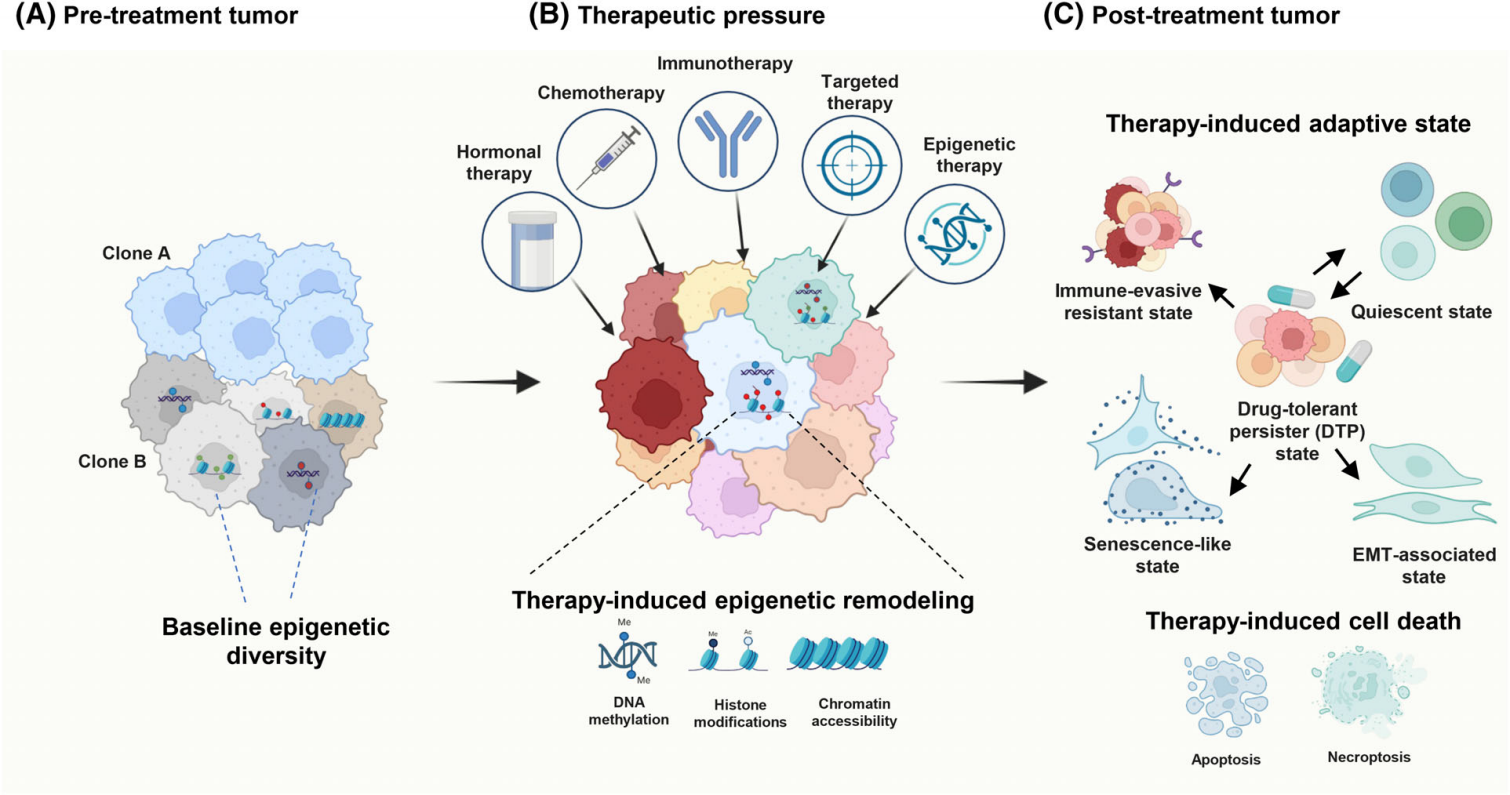
Therapy‐induced epigenetic remodeling and cellular state adaptation. This framework illustrates how epigenetic plasticity, rather than genetic selection alone, drives therapy‐induced cellular adaptation and resistance. (A) Tumors exhibit extensive genetic and epigenetic heterogeneity before treatment. (B) Diverse therapeutic pressures are associated with dynamic epigenetic remodeling across multiple layers, including DNA methylation, histone modifications, and chromatin accessibility, which may contribute to adaptive transcriptional reprogramming in a context‐dependent manner. (C) Post‐treatment tumors display heterogeneous cellular states. Although a subset of cells undergoes programmed cell death, including apoptosis or necroptosis, surviving populations may adopt diverse adaptive phenotypes, including drug‐tolerant persisters (DTPs), quiescent cells, epithelial–mesenchymal transition (EMT)‐associated states, immune‐evasive populations, and senescence‐like cells.

### 2.1 Pretreatment heterogeneity establishes the landscape for therapy‐induced adaptation

Pretreatment tumors exhibit substantial epigenetic heterogeneity which shapes the diversity of subsequent therapeutic responses. Single‐cell analyses have demonstrated that transcriptional and epigenetic variability arise even among cancer cells with highly similar mutational profiles [14, 15]. At the molecular level, this variability is driven by cell‐to‐cell differences in chromatin accessibility and cis‐regulatory element activity, which establish distinct transcriptional programs independently of underlying mutational status [16, 17] (Fig. 1A). In addition, single‐cell RNA sequencing studies have revealed that pretreatment tumors contain a diverse phenotypic spectrum characterized by transcriptional programs associated with proliferation, invasiveness, stem cell properties, and differentiation [18, 19]. Moreover, epigenetic heterogeneity represents functionally meaningful variations, as distinct cell states exhibit inherently different sensitivities to therapeutic agents [20]. For instance, cells in a quiescent state with slow cell cycle kinetics were less responsive to chemotherapies [21, 22]. Conversely, cells with transient transcriptional upregulation of survival‐associated programs exhibit reduced susceptibility to targeted therapies [23]. Overall, these pre‐existing cellular states establish a heterogeneous baseline from which adaptive epigenetic reprogramming is driven under therapeutic pressure.

### 2.2 Diverse therapeutic pressure promotes epigenetic reprogramming

Therapeutic intervention reshapes the epigenetic landscape of tumor cells, contributing to adaptive cellular responses and treatment resistance (Fig. 1B, Table 1) [24]. Although cytotoxic and targeted therapies act through distinct molecular mechanisms, both converge on epigenetic remodeling programs that facilitate cell state transitions. In response to chemotherapy, surviving cells undergo widespread epigenetic alterations, including changes in DNA methylation and chromatin organization [25]. DNA damage responses lead to additional changes in histone modification patterns and promote heterochromatin remodeling, thereby activating previously silenced gene programs [26, 27]. Similarly, targeted therapies that block oncogenic signaling pathways activate compensatory epigenetic mechanisms in response to signaling deprivation [28]. For instance, in lung cancer cells treated with epidermal growth factor receptor (EGFR) inhibitors, increased activity of chromatin remodelers enhances chromatin accessibility to genes encoding alternative receptor tyrosine kinases, facilitating the activation of bypass signaling pathways [29, 30]. Additionally, targeted therapy reprograms histone modification patterns in genes involved in cell fate determination through treatment‐induced alterations in transcription factor (TF) occupancy [31]. Collectively, these therapies converge on epigenetic reprogramming as a shared mechanism of adaptive survival. Immunotherapy, hormonal therapy, and epigenetic drugs impose additional selective pressures via distinct but interconnected mechanisms.

**Table 1:** Studies on therapy‐induced epigenetic plasticity.

Therapy class	Cancer type	Example therapy	Key epigenetic remodeling	Associated adaptive state	References
Chemotherapy	Triple‐negative breast cancer	Capecitabine (5‐FU)	Demethylation of H3K27me3	DTP^a^	[25]
Chemotherapy	Neuroblastoma	Cyclophosphamide‐based	Remodeling of chromatin accessibility	DTP, EMT‐like^b^	[27]
Chemotherapy	Lung cancer	Doxorubicin	Chromatin remodeling	Senescence‐like^c^	[49]
Chemotherapy/Targeted therapy	Multiple cancer types	Multiple cytotoxic agents (Paclitaxel, Erlotinib)	Demethylation of H3K4me3	DTP	[26]
Targeted therapy	EGFR‐mutant NSCLC	Osimertinib	Chromatin accessibility reprogramming	DTP, EMT‐like	[29]
Targeted therapy	EGFR‐mutant NSCLC	Osimertinib	Chromatin accessibility reprogramming	DTP	[30]
Targeted therapy	BRAF‐mutant melanoma	BRAF inhibitor (Vemurafenib)	Histone modification changes	EMT‐like	[31]
Immunotherapy	Multiple solid tumors	Immune checkpoint blockade	SETDB1‐mediated epigenetic silencing	Immune‐evasive state^d^	[32]
Immunotherapy	Esophageal squamous cell carcinoma	Immune pressure (tumor microenvironment)	Promoter DNA hypermethylation	Immune‐evasive state	[33]
Immunotherapy	Melanoma	Immune checkpoint blockade	DNA methylation–dependent regulation	Immune‐evasive state	[34]
Hormonal therapy	Breast cancer	Endocrine therapy (tamoxifen, aromatase inhibitors)	DNA methylation and repressive histone modifications	DTP, Dormant/quiescent^e^	[36]
Hormonal therapy	Breast cancer	Endocrine therapy	Epigenetic reprogramming at estrogen receptor binding sites	DTP, EMT‐like	[39]
Hormonal therapy	Prostate cancer	AR‐targeted therapy (enzalutamide)	Chromatin accessibility reprogramming	EMT‐like	[37]
Epigenetic therapy	Colorectal cancer	DNMTi (decitabine)	Demethylation of transposable elements	Immune‐evasive state	[40]
Epigenetic therapy	Multiple cancer types	DNMT inhibitors	DNA hypomethylation, accumulation of H3K27me3	DTP	[43]

a Drug‐tolerant persister (DTP): A rare population of cancer cells that survives therapy through reversible, nongenetic adaptive mechanisms without requiring stable resistance‐conferring mutations.

b EMT‐like: A plastic cell state in which tumor cells lose epithelial traits and acquire mesenchymal features, often along a continuum of partial or hybrid states rather than a binary switch.

c Senescence‐like state: A therapy‐associated state of stable cell cycle arrest in which cells remain metabolically active and often acquire a senescence‐associated secretory phenotype (SASP).

d Immune‐evasive state: A tumor cell state that enables escape from immune elimination through reduced antigen presentation and/or increased immunosuppressive signaling.

e Dormant/quiescent state: A reversible nonproliferative or slow‐cycling state in which tumor cells remain viable and retain the capacity to re‐enter the cell cycle.

Immunotherapy augments immune pressure within the tumor microenvironment, driving epigenetic adaptation in tumor cells, which is characterized by downregulation of the antigen presentation machinery and upregulation of immune evasion‐associated genes [32]. For instance, major histocompatibility complex (MHC) genes can be silenced through promoter DNA hypermethylation, whereas immune checkpoint ligands can be upregulated through chromatin remodeling at their regulatory regions [33, 34, 35]. Hormonal therapy, particularly in breast and prostate cancers, triggers chromatin structural changes in hormone receptor genes and their downstream targets, with increased DNA methylation or accumulation of repressive histone modifications at enhancer regions, ultimately enabling hormone‐independent transcriptional programs [36]. These processes involve the extensive epigenetic reprogramming of regulatory elements. These include aberrant enhancer–promoter interactions in endocrine‐resistant breast cancer and reprogramming of androgen receptor (AR) chromatin binding to developmental gene programs in therapy‐resistant prostate cancer [37, 38, 39]. Furthermore, epigenetic agents can induce unintended chromatin restructuring beyond their intended targets. Specifically, DNA methyltransferase inhibitor‐mediated demethylation of transposable elements triggers induction of endogenous retroviral sequences and activates innate immune signaling dependent on melanoma differentiation‐associated protein 5 and mitochondrial antiviral‐signaling protein [40, 41]. Paradoxically, this process simultaneously induces compensatory H3K27me3 accumulation at newly demethylated genomic regions, establishing an adaptive transcriptional state that can confer cross‐resistance to subsequent treatments [42, 43]. This interconnected epigenetic reprogramming enables rapid transitions to survival‐advantaged cell states, establishing the molecular basis for adaptive phenotypic transitions.

### 2.3 Epigenetic plasticity diversifies therapy‐adaptive cell states

Therapy‐induced epigenetic remodeling generates phenotypically distinct cellular states that represent the major mechanisms of treatment failure. Although most treatment‐sensitive cells undergo apoptosis, a minor population of cells is characterized by pre‐existing or therapy‐induced epigenetically driven transitions into adaptive states that promote survival and therapeutic resistance (Fig. 1C) [44, 45, 46]. These surviving cells evade therapeutic elimination through epigenetics‐driven phenotypic reprogramming rather than through stable genetic mutations, representing a significant nongenetic mechanism of treatment resistance.

Multiple distinct adaptive states were observed following therapeutic exposure (Fig. 1C). The DTP state is induced during the early treatment phases and is characterized by slow cell cycle progression, activation of stress response pathways, and widespread chromatin remodeling associated with a reversible, epigenetically regulated transcriptional program [45, 47]. The senescence‐like state exhibits stable cell cycle arrest while maintaining metabolic activity and actively remodels the tumor microenvironment through pro‐tumorigenic senescence‐associated secretory phenotype (SASP) signaling [48, 49, 50]. Epithelial–mesenchymal transition (EMT)‐associated reprogramming, driven by epigenetic silencing of epithelial identity genes and coordinated upregulation of mesenchymal transcription factors (TFs), confers enhanced invasive and metastatic potential while promoting cross‐resistance to multiple therapeutic modalities [51]. The immune‐evasive state enables escape from immune recognition and represents a major barrier to immunotherapy efficacy, arising from epigenetic silencing of the antigen presentation machinery and upregulation of immune checkpoint ligands [52, 53]. The quiescent state confers resistance to proliferation‐dependent therapeutic agents through minimal metabolic activity and cell cycle arrest maintained by repressive chromatin states [54, 55].

These adaptive states frequently coexist, and clear boundaries between them are rarely observed in clinical tumors. Individual cells can exhibit the characteristics of more than one state simultaneously or can transition between states in response to changes in treatment conditions and the tumor microenvironment [45, 47]. For example, DTP cells may acquire senescence‐like characteristics or EMT‐related programs over time, whereas quiescent cells may re‐enter the cell cycle to drive tumor relapse [54]. Critically, these state transitions are influenced by epigenomic reprogramming, and the duration and reversibility of each state depend on the degree of chromatin remodeling and the persistence of external therapeutic pressure [56]. Therefore, epigenetic plasticity functions as a mechanism for short‐term survival. It also drives progressive intratumoral diversification, highlighting the need for therapeutic strategies that target the epigenetic basis of state transitions rather than the individual resistant states alone [56].

## 3 Key epigenetic mechanisms enabling adaptive plasticity under therapy

The diverse adaptive cellular states induced by therapeutic interventions are supported by fundamental epigenetic mechanisms that collectively reshape the regulatory landscape of tumor cells. Among the major regulatory layers involved, DNA methylation reprogramming, chromatin accessibility dynamics, and histone modification remodeling are prominently implicated in cellular plasticity (Fig. 2) [24, 57]. Understanding the individual and interactive functions of these mechanisms provides critical insights into the rapid adaptation of cancer cells to therapeutic pressures without requiring stable genetic mutations. Although these regulatory layers are discussed separately for clarity, they often interact in biological settings and may contribute to adaptive phenotypes in a context‐dependent manner. Accordingly, Fig. 2 presents these relationships as overlapping and nonexclusive regulatory patterns, with similar cellular phenotypes arising through different epigenetic routes and related epigenetic alterations converging on overlapping adaptive states.

**Fig. 2:**
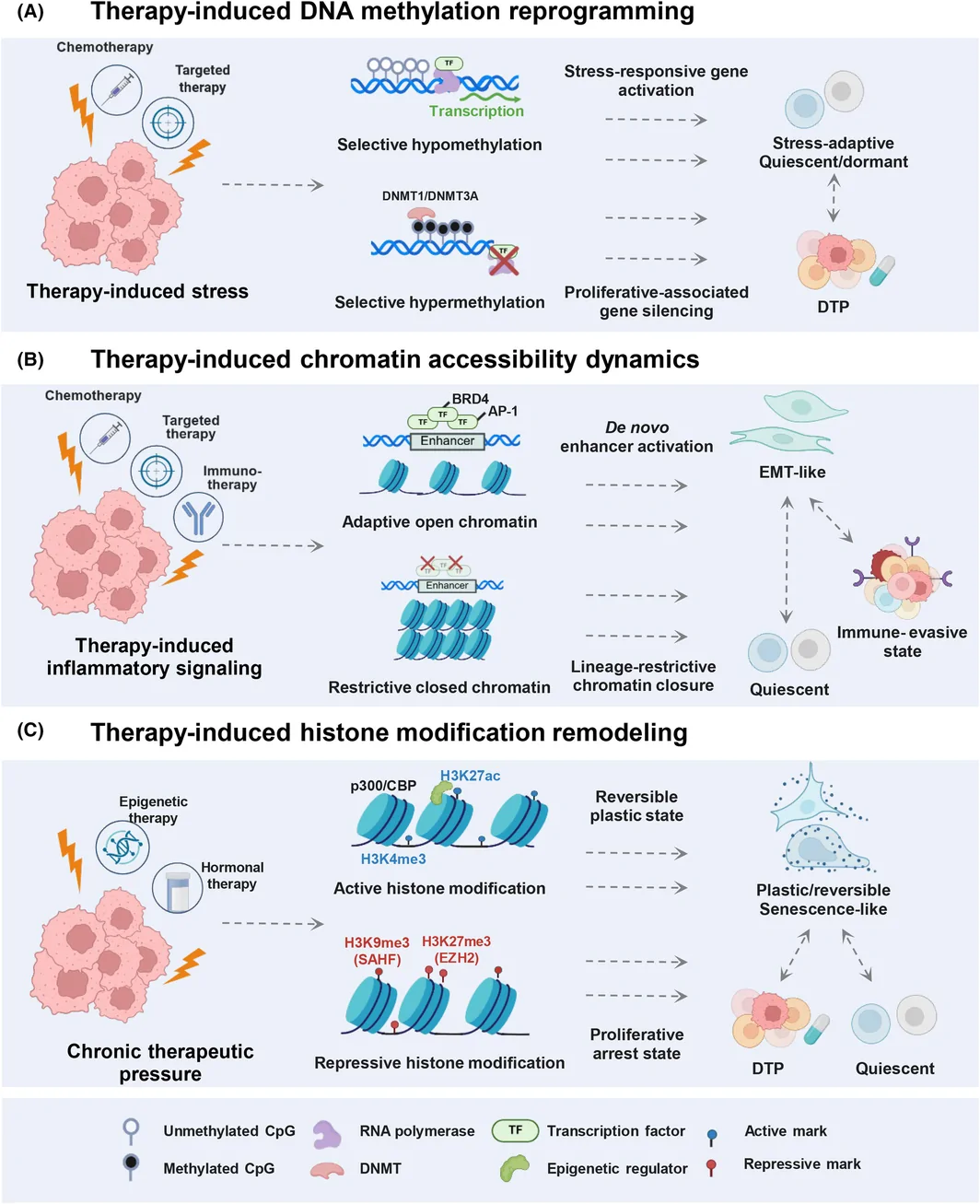
Epigenetic regulatory mechanisms underlying therapy‐induced cell states. Schematic representation of major epigenetic mechanisms implicated in therapy‐induced cellular adaptation. (A) Therapeutic pressure is associated with context‐dependent DNA methylation reprogramming, including selective hypomethylation at stress‐responsive loci and DNA methyltransferase 1 (DNMT1) or DNA methyltransferase 3 alpha (DNMT3A)‐mediated hypermethylation at proliferative loci, with distinct functional consequences for survival and growth‐associated gene programs. (B) Therapy‐induced signaling can reshape chromatin accessibility through context‐dependent transcription factor activity, including bromodomain‐containing protein 4 (BRD4) and activator protein 1 (AP‐1)‐associated enhancer activation, thereby contributing to adaptive transcriptional reprogramming across diverse cellular states. (C) Sustained therapeutic stress is associated with dynamic alterations in the histone modification landscape, encompassing both active marks such as histone H3 lysine 27 acetylation (H3K27ac) and histone H3 lysine 4 trimethylation (H3K4me3) and repressive marks such as histone H3 lysine 9 trimethylation (H3K9me3) and histone H3 lysine 27 trimethylation (H3K27me3), which may support diverse and partially overlapping adaptive phenotypes. These relationships may differ by context rather than follow a single fixed pattern, and overlapping mechanisms can be observed across different tumor types and therapeutic settings.

### 3.1 DNA methylation reprogramming

DNA methylation, which is characterized by the covalent addition of a methyl group to cytosine bases at CpG sites, is one of the most stable epigenetic modifications under baseline cellular conditions. However, therapeutic stress can induce widespread or loci‐specific alterations in DNA methylation patterns, thereby affecting gene expression (Fig. 2A). These therapy‐induced changes occur through two opposing processes, selective hypomethylation and selective hypermethylation, each driving distinct adaptive responses [58, 59].

#### 3.1.1 Selective hypomethylation and stress‐responsive gene activation

Therapeutic pressure induces strong metabolic stress in cells, leading to transcriptional activation of stress response genes as an adaptive survival mechanism. Key stress response genes, such as CDKN1A and GADD45A, were rapidly activated after treatment, and their expression was regulated by epigenetic changes, including DNA demethylation and histone modifications in the promoter and enhancer regions [60, 61]. Chemotherapy‐induced DNA damage has been associated with DNA repair pathways, in part through demethylation of repair gene promoters, whereas targeted therapies have been linked to hypomethylation of alternative survival pathway genes, enabling bypass signaling [58, 62]. Drug‐induced hypomethylation involves passive and active mechanisms. Passive demethylation occurs under stressful conditions when DNA methyltransferases (DNMTs) fail to maintain the methylation marks during DNA replication. Active DNA demethylation is mediated by ten‐eleven translocation (TET) enzymes that oxidize 5‐methylcytosine to form an intermediate that is subsequently removed through base excision repair [63]. Under sustained therapeutic pressure, the integrated stress response (ISR) pathway was also activated. ISR components such as ATF4, ATF3, and TRIB3 are upregulated through translational and epigenetic mechanisms, and these signals are closely linked to cell cycle arrest and the transition to a dormant state [64, 65]. During this process, genes involved in cell cycle arrest, autophagy, and metabolic reprogramming may undergo demethylation‐associated activation, contributing to reduced proliferation and entry into a temporary quiescent‐like state [66, 67]. These epigenetically regulated dormant/quiescent cells exhibit increased chromatin condensation and decreased overall transcriptional activity. This epigenetic state is reversible and results in rapid transcriptional activation in response to microenvironmental signals and treatment withdrawal.

#### 3.1.2 Selective hypermethylation and proliferative gene silencing

Therapeutic stress induces selective hypermethylation in tumor cells by inducing the aberrant binding of DNMT1 and DNMT3A/3B to promoter CpG islands. This binding is often mediated by stress‐activated TFs or chromatin remodeling complexes [62, 68]. Consequently, key tumor suppressor and proliferation‐regulating genes were epigenetically silenced. Hypermethylation of genes such as CDKN2A and MLH1 suppressed apoptotic signaling and impaired mismatch repair, thereby disrupting cell cycle regulation and promoting survival under sustained treatment conditions [69, 70]. Simultaneously, the hypermethylation of pro‐apoptotic gene promoters further reduced apoptotic responses, thereby enabling cells to survive repeated treatment cycles [71]. These hypermethylation events provide an epigenetic basis for various adaptive cellular states observed during treatment. In DTP cells, epigenetic silencing of genes regulating proliferation and lineage differentiation, mediated through chromatin‐level reprogramming including histone modification and DNA methylation changes, has been associated with a phenotype characterized by slow cell cycle kinetics and stress tolerance [7, 71, 72]. This state provides the cells with resistance to both chemotherapy and targeted therapy. By contrast, a more repressed dormant or quiescent state is maintained through complex interactions between DNA hypermethylation and repressive histone modifications [7, 25]. Cooperative gene silencing suppresses cell growth, while preserving the ability to proliferate upon treatment withdrawal or environmental changes. This cooperative gene silencing is a key driver of therapy‐induced epigenetic adaptation and reversible tumor cell survival.

### 3.2 Chromatin accessibility dynamics

Chromatin accessibility describes the availability of regulatory DNA elements for interactions with TFs and associated regulators. Accessibility is determined by nucleosome positioning, chromatin compaction, and chromatin remodeling activity (Fig. 2B). Unlike DNA methylation and histone modification, chromatin accessibility is considered to respond more rapidly to environmental and therapeutic signals.

#### 3.2.1 Adaptive open chromatin formation and enhancer activation

Therapeutic pressure promotes the formation of accessible chromatin at previously closed genomic loci in tumor cells, thereby enabling the activation of adaptive gene expression programs. These changes are mediated by the recruitment of ATP‐dependent chromatin remodeling complexes. The SWI/SNF, ISWI, and CHD family complexes reorganize chromatin structure by rearranging or removing nucleosomes [73, 74]. A central feature of this process is *de novo* activation of enhancers that drive alternative cell fate programs. Under therapeutic stress, stress‐responsive TFs such as activator protein‐1 (AP‐1) bind to newly accessible regulatory regions and recruit chromatin remodeling complexes to establish adaptive enhancer activity. These therapy‐induced enhancer remodeling events frequently activate developmental or lineage plasticity programs [75, 76]. For instance, chemotherapy and targeted therapies activate enhancers associated with EMT, promoting a mesenchymal and invasive phenotype. This transition involves increased promoter DNA methylation of genes that maintain epithelial identity and the activation of enhancers for gene programs associated with mesenchymal traits. The chromatin remodeling complex SWI/SNF, particularly its BRG1 (SMARCA4) subunit, played a critical role in establishing accessible chromatin at EMT‐associated enhancers, while p300 and CBP stabilized accessibility by depositing histone acetylation marks on newly opened enhancers [77, 78]. These alterations drive increased expression of EMT‐associated TFs such as ZEB1, SNAIL, and TWIST, promoting a mesenchymal‐like phenotype associated with therapeutic resistance and metastatic potential [79]. Persistent immune pressure induced the opening of chromatin regions associated with immune checkpoint ligands and immunosuppressive pathways, leading to immune‐evasive resistance. Cancer cells evade immune recognition through epigenetic silencing of MHC class I genes, increased surface expression of immune checkpoint molecules (PD‐L1), and downregulation of antigen presentation mechanisms. These alterations are primarily mediated by coordinated changes in chromatin accessibility and histone modifications [80, 81].

#### 3.2.2 Restrictive chromatin closure and lineage‐specific silencing

Therapeutic stress promotes closure of chromatin regions originally associated with cellular identity or proliferative programs. Chromatin condensation involves the deposition of repressive histone modifications, recruitment of heterochromatin proteins, and the exclusion of TFs from previously accessible regulatory elements [25, 82]. Lineage‐restricted chromatin closure is a key mechanism enabling cellular dedifferentiation under therapy. In prostate cancer treated with AR inhibitors, long‐term treatment closed AR‐bound enhancers and opened the enhancers associated with neuroendocrine differentiation. This remodeling facilitated the transition to AR‐independent phenotypes [83, 84]. Similarly, in melanomas treated with BRAF inhibitors, chromatin accessibility shifted from melanocytic lineage programs to a neural crest‐like state [85]. Thus, chromatin closure and repressive epigenetic remodeling beyond transcriptional silencing events serve as active drivers of lineage reprogramming and therapeutic adaptation.

### 3.3 Histone modification remodeling

Histone modifications are diverse post‐translational modifications of histone proteins that influence chromatin organization and transcriptional activity. In contrast to the relative stability of DNA methylation, histone modifications are highly dynamic and reversible, enabling rapid transcriptional responses to therapeutic stress (Fig. 2C) [86].

#### 3.3.1 Active histone modifications and reversible plastic states

A transcriptionally permissive chromatin state is established by the activation of histone modifications, with H3K4me3 and H3K27ac serving as hallmarks of acetylation‐ and methylation‐driven gene activation. Therapeutic pressure alters the genomic distribution of these marks by regulating histone acetyltransferases (HATs) and methyltransferases including p300/CBP [87]. Under therapeutic stress, cells exhibited an increased accumulation of active histone marks at stress response genes, alternative survival pathway genes, and developmental TF loci. H3K27 acetylation became redistributed to newly activated enhancer elements, thereby driving adaptive cell states. Bromodomain and extraterminal (BET) family proteins recognize acetylated histones, stabilize active chromatin states, and recruit transcriptional machinery [88].

Therapy‐induced changes in active histone modifications contribute to a reversible state of cellular plasticity. Unlike permanent genetic changes, histone acetylation and methylation can be rapidly performed or removed by opposing enzymatic activities. This reversibility explains why resistant cell populations revert to drug‐sensitive states after treatment withdrawal. Senescence‐like cells may exhibit features of this reversible state, displaying characteristic histone modification patterns, including H3K9me3 redistribution and the formation of senescence‐associated heterochromatin foci [89]. These cells exhibited cell cycle arrest while maintaining metabolic activity and remodeling the tumor microenvironment through SASP [90]. Their chromatin exhibited increased heterochromatic foci and alterations in DNA methylation at the promoters of cell cycle regulators such as CDKN1A and CDKN2A. Under SASP‐mediated microenvironmental signaling or treatment withdrawal, a subset of these cells regained their proliferative capacity, indicating retained epigenetic plasticity.

#### 3.3.2 Repressive histone modifications and dormant states

Repressive histone modifications, including H3K9me3, H3K27me3, and H4K20me3, form transcriptionally inactive chromatin regions. Therapeutic pressure promotes the accumulation of these repressive marks on genes involved in proliferation and differentiation, thereby facilitating entry into quiescent or dormant states [91]. H3K27me3 is a repressive histone mark catalyzed by polycomb repressive complex 2 (PRC2), which includes the methyltransferase EZH2, and is particularly important in therapy‐induced adaptation [92]. Paradoxically, epigenetic and hormonal therapies increased EZH2 activity at the promoters of lineage‐specific TFs such as GATA3 and FOXA1, suppressing differentiation programs and promoting lineage reprogramming. This repressive chromatin environment maintains slow‐cycling and drug‐tolerant states that persist throughout treatment. H3K9me3, deposited by SUV39H1/2 and SETDB1 methyltransferases, forms constitutive and alternative heterochromatin [93]. Therapeutic stress triggered the redistribution of H3K9me3, establishing new heterochromatin regions that repressed proliferation‐associated genes. These H3K9me3‐marked regions often overlap with DNA methylation, generating reinforced repressive chromatin states [82].

### 3.4 Therapy‐specific signatures driven by multiple epigenetic crosstalk

Recent evidence indicates that therapy‐induced epigenetic responses share both common adaptive programs and modality‐specific signatures. Dissecting these convergent and divergent epigenetic patterns is essential for understanding the broader principles of therapeutic resistance and identifying potential vulnerabilities for treatment.

#### 3.4.1 Common epigenetic programs across therapies

Epigenetic responses to chemotherapy, targeted therapy, immunotherapy, and hormonal therapy mechanistically lead to shared adaptive strategies despite their distinct modes of action [94, 95]. A notable mechanism was the demethylation of AP‐1 and NF‐κB binding motifs across diverse therapeutic pressures, enabling broad activation of stress‐responsive transcriptional programs [23, 96, 97]. Thus, stress‐sensing TF networks may be a common epigenetic entry point through which cancer cells initiate adaptive reprogramming, regardless of the treatment modality. Additionally, DNA methylation and chromatin condensation suppress lineage‐specific TF programs, enabling tumor cells to escape their pre‐existing differentiation state [98, 99]. Consequently, cells acquire the epigenetic plasticity necessary for phenotypic transitions, which is a common adaptive strategy used in therapeutic settings.

#### 3.4.2 Therapy‐specific epigenetic signatures and cross‐layer integration

In addition to these shared responses, each treatment exhibits a distinct epigenetic signature arising from its unique mechanism of action. Chemotherapy induced phosphorylation of H2AX (γH2AX) and extensive chromatin remodeling at DNA damage sites, establishing a damage‐related epigenetic landscape that was rarely observed in other treatments [100, 101]. Immunotherapy generated unique epigenetic signatures driven by interferon signaling. These signatures included enhancer‐mediated upregulation of immune checkpoint ligands through enhancer remodeling and downregulation of antigen‐presenting genes via promoter hypermethylation. Thus, tumors may adapt to immune pressure [102, 103]. Epigenetic therapies displayed paradoxical molecular responses. DNA methyltransferase inhibitors reduced DNA methylation but simultaneously induced compensatory increases in repressive histone modifications, including H3K27me3 and H3K9me3, at the promoters of genes involved in cell survival and proliferation [104].

These therapy‐associated signatures arise from multiple epigenetic alterations, specifically the coordinated interactions between DNA methylation, histone modifications, and chromatin accessibility. Therapy‐induced changes in a single layer propagate through epigenetic networks. Damage‐responsive histone modifications recruited DNA methyltransferases to reinforce this repressive state. Altered chromatin accessibility has been shown to influence the recruitment of histone‐modifying enzymes to defined genomic loci. This epigenetic crosstalk may explain how distinct therapeutic pressures generate phenotypically diverse resistant populations [105]. Targeting context‐dependent epigenetic vulnerabilities may offer more precise strategies for overcoming adaptive resistance. This approach focuses on shared regulatory states rather than individual resistance mechanisms.

## 4 Single‐cell multi‐omics technologies for therapy‐induced epigenetic plasticity profiling

Single‐cell multi‐omics technologies have substantially advanced our ability to resolve the epigenetic and transcriptional heterogeneity underlying therapy‐induced tumor adaptation. Conventional bulk sequencing approaches mask rare and transitional cell populations that play a pivotal role in driving therapeutic resistance by averaging molecular signals across millions of cells. By simultaneously capturing multiple molecular layers at a single‐cell resolution, these approaches enable the direct investigation of how epigenetic states translate into distinct transcriptional programs across diverse cellular populations (Fig. 3). An effective study design requires a clear understanding of platform principles and integration strategies. This helps to achieve a more accurate analysis of therapy‐induced epigenetic plasticity.

**Fig. 3:**
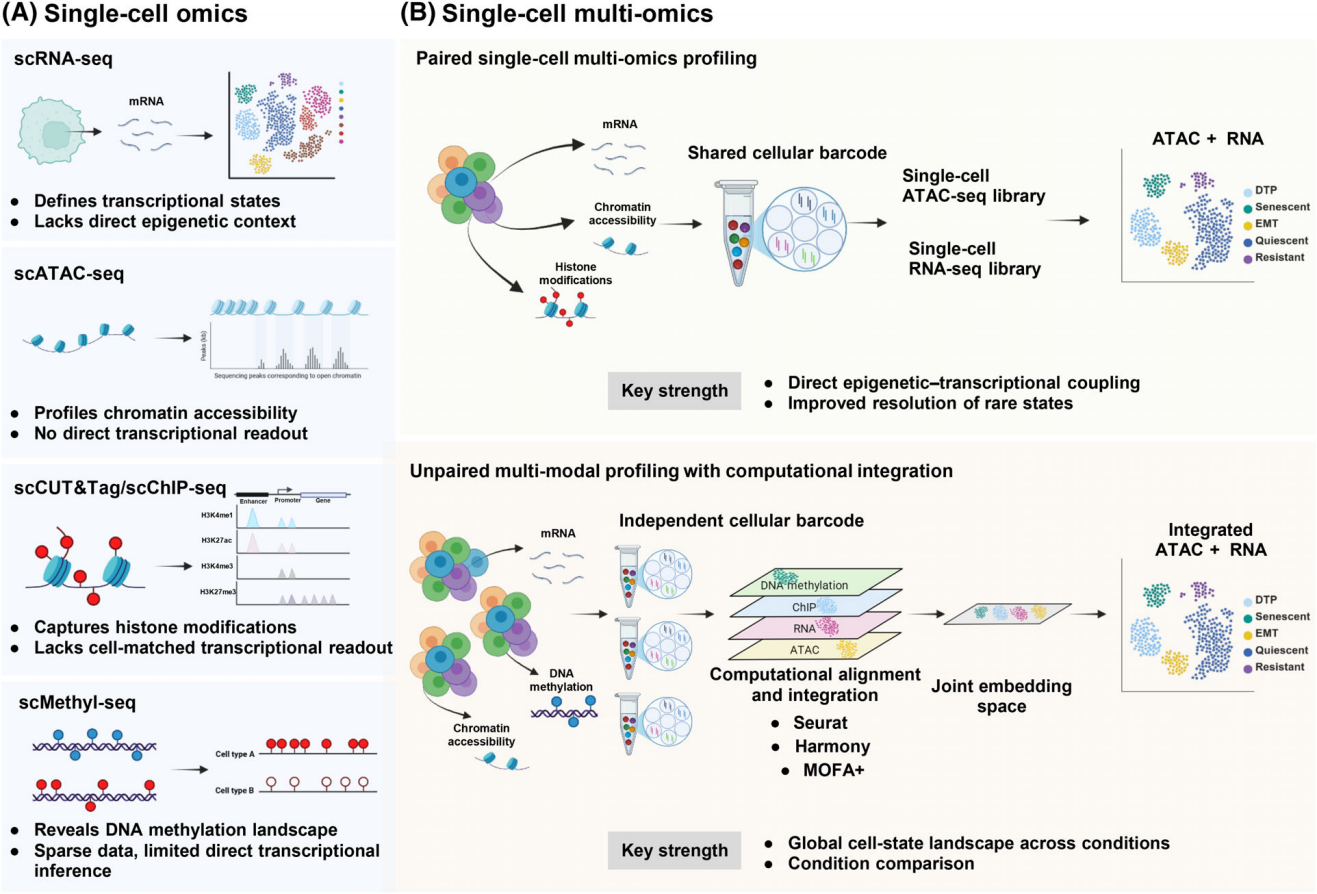
Single‐cell omics and multi‐omics approaches to resolve therapy‐induced epigenetic plasticity. Schematic overview of single‐cell and multi‐omics strategies for dissecting therapy‐induced epigenetic plasticity across molecular layers. (A) Individual single‐cell platforms profile distinct molecular layers: single‐cell RNA sequencing (scRNA‐seq) captures transcriptomes, single‐cell assay for transposase‐accessible chromatin using sequencing (scATAC‐seq) measures chromatin accessibility, single‐cell cleavage under targets and tagmentation (scCUT&Tag)/single‐cell chromatin immunoprecipitation sequencing (scChIP‐seq) detects histone modifications, and single‐cell methylome sequencing (scMethyl‐seq) reveals DNA methylation patterns. Each platform provides unique insights but lacks integrated multimodal information. (B) Integration strategies overcome single‐modality limitations. Paired approaches, including 10× Multiome and simultaneous high‐throughput assay for transposase‐accessible chromatin and RNA expression with sequencing (SHARE‐seq), simultaneously profile ribonucleic acid (RNA) and chromatin accessibility in the same cells using shared barcodes, enabling direct coupling of epigenetic and transcriptional states. Unpaired approaches computationally integrate separately profiled modalities (scRNA‐seq, scATAC‐seq, scMethyl‐seq) using methods such as Seurat, Harmony, and Multi‐Omics Factor Analysis Plus (MOFA+) to generate unified cell state landscapes for comparative analysis across treatment conditions.

### 4.1 Single‐cell omics platforms to investigate therapy‐induced tumor adaptation

Single‐cell omics platforms can be broadly categorized according to their molecular layers, thereby providing complementary views of the cellular state (Fig. 3A). Single‐cell RNA sequencing (scRNA‐seq) remains the most widely adopted platform for defining transcriptional states at a single‐cell resolution. By capturing polyadenylated mRNA from individual cells, scRNA‐seq enables the high‐resolution identification of cell populations, trajectory inference, and gene regulatory network analysis. To analyze therapy‐induced plasticity, scRNA‐seq was used to reveal dynamic phenotypic changes in the evolution of drug resistance. For instance, in non‐small cell lung cancer, short‐term exposure to alectinib induced rapid transcriptional state shifts, indicating direct drug‐induced cellular adaptation [106, 107]. Furthermore, a patient‐derived xenograft model of EGFR‐mutant lung adenocarcinoma identified a rare subpopulation of DTP‐like cells. These cells represented approximately 4% of basal tumor cells and displayed transcriptomic features similar to DTP cells [108]. However, scRNA‐seq alone remains limited in its capacity to directly capture the epigenetic features that shape the observed gene expression programs [16].

Single‐cell assay for transposase‐accessible chromatin sequencing (scATAC‐seq) profiles genome‐wide chromatin accessibility. This enabled the identification of active regulatory elements, TF‐binding motifs, and chromatin remodeling events associated with cell state transitions. The integration of scRNA‐seq and scATAC‐seq has proven to be particularly powerful for resolving therapy‐induced epigenetic heterogeneity. In prostate cancer, one study applied both modalities to an LNCaP‐derived cell‐line model of enzalutamide treatment response and resistance. This analysis identified pre‐existing and treatment‐persistent cell subpopulations with distinct chromatin landscapes linked to alternative transcriptional programs. The study also showed increased chromatin accessibility in persister‐like populations following AR‐targeting therapy [109]. Similarly, integrated scRNA‐seq and scATAC‐seq analyses profiled over 80 000 cells from primary and recurrent tamoxifen‐resistant breast tumors. This study identified distinct epigenetically regulated cancer cell states enriched in endocrine‐resistant tumors [20]. Despite these strengths, scATAC‐seq provides no direct transcriptional readout and requires computational inference to link accessibility changes to gene expression.

Single‐cell cleavage under targets and tagmentation (scCUT&Tag) and single‐cell chromatin immunoprecipitation sequencing (scChIP‐seq) extend epigenomic profiling to histone modifications, enabling genome‐wide mapping of marks such as H3K27ac, H3K4me3, H3K27me3, and H3K9me3 [25, 110]. These approaches have been directly applied to dissect therapy‐induced epigenetic plasticity in cancers. In breast cancer, scChIP‐seq profiling of H3K27me3 revealed that a minor fraction of cells within drug‐sensitive tumors harbored chromatin signatures characteristic of therapy‐resistant states prior to treatment. These findings suggest that histone modification heterogeneity may underlie pre‐existing resistance potential [17]. These approaches are valuable for resolving bivalent chromatin states and repressive histone modification patterns associated with therapy‐induced dormancy and DTP phenotypes. A key limitation is the absence of cell‐matched transcriptional readout, which limits direct epigenetic–transcriptional coupling within the same cell.

Single‐cell methylation sequencing approaches enable genome‐wide profiling of DNA methylation at single‐cell resolution [111, 112]. The application of these approaches in therapeutic contexts has revealed dynamic DNA hypo‐ and hypermethylation events associated with adaptive gene expression reprogramming [113]. Despite their ability to resolve cell‐to‐cell methylation heterogeneity, these approaches generate characteristically sparse data because of limited CpG coverage per cell, thereby limiting direct transcriptional inference. In cancer, these approaches have further shown that therapy‐driven epigenetic evolution can occur through clonally heritable DNA methylation changes, as demonstrated in chronic lymphocytic leukemia (CLL). Single‐cell methylome profiling before and during ibrutinib treatment revealed lineage dynamics and transcriptional heterogeneity linked to elevated epimutation rates [114]. In glioblastoma, integrated single‐cell methylome and transcriptomic analyses indicated that methylation landscapes were associated with transcriptional cell states exhibiting distinct degrees of heritability and plasticity under therapy [115].

Single‐cell proteomics represents a promising complement to transcriptomic and epigenomic approaches by enabling the direct profiling of protein abundance and proteomic heterogeneity at single‐cell resolution. Mass spectrometry‐based methods, such as SCoPE2 and nanoPOTS, have quantified hundreds to thousands of proteins per cell, revealing that proteomic heterogeneity exceeds the resolution afforded by transcriptional profiling alone [116, 117]. However, substantial technical barriers, including limited protein material per cell, the lack of a PCR‐equivalent amplification strategy, and insufficient throughput and standardization, currently limit large‐scale applications. Integration with transcriptomic and epigenomic data remains an important future direction [118, 119].

### 4.2 Multi‐omics integration strategies for therapeutic adaptation

Although individual single‐cell platforms provide valuable information on diverse cell states, each capture only a single molecular layer and offers only a partial view of the regulatory mechanisms. Integrating multiple molecular layers within or across single cells has enabled a more comprehensive understanding of the epigenetic mechanisms that drive transcriptional reprogramming under therapeutic conditions (Fig. 3B). Two principal strategies are typically employed to achieve this integration. Paired approaches simultaneously measure multiple modalities within the same cell, whereas unpaired approaches computationally align the profiled datasets independently.

#### 4.2.1 Paired single‐cell multi‐omics approaches

Paired multi‐omics approaches simultaneously capture two or more molecular modalities from the same individual cell using a shared cell barcode, enabling direct within‐cell coupling of epigenetic and transcriptional information [11]. The 10× Genomics Chromium Single‐Cell Multiome platform, which jointly profiles chromatin accessibility and gene expression in the same nucleus, represents the most widely adopted paired approach. Simultaneous high‐throughput ATAC and RNA expression sequencing (SHARE‐seq) enables simultaneous measurement of chromatin accessibility and transcriptome within individual cells [120]. This approach has been applied to demonstrate that chromatin accessibility preceded and predicted transcriptional cell fate decisions, thereby establishing a relationship between epigenetic and transcriptional states [120, 121, 122].

The defining strength of paired approaches lies in their ability to establish direct epigenetic–transcriptional coupling at single‐cell resolution. The 10× Multiome platform has been applied in a longitudinal analysis of matched primary and recurrent tumors in MYC‐driven medulloblastoma. This analysis identified metabolism‐linked epigenetic reprogramming as a driver of therapy resistance and revealed a persistent progenitor population characterized by IDH1‐mediated metabolic and epigenetic switching [123]. In multiple myeloma, single‐nucleus multi‐omics analysis has been applied in clinical trials. Genetic inactivation and epigenetic silencing of regulatory elements have been identified as concurrent mechanisms of resistance to monoclonal antibody therapy [124]. In colorectal cancer patient‐derived organoids, longitudinal lineage tracking combined with single‐cell multi‐omics revealed that distinct subclonal populations were selected by different targeted therapies [125]. The cellular memory of drug resistance was encoded as a heritable epigenetic configuration from which multiple transcriptional programs could arise. These findings supported a one‐to‐many epigenotype‐to‐phenotype map that jointly accounted for clonal expansion and transcriptional plasticity [125]. Paired approaches substantially improve the detection of rare and heterogeneous cell states and enable the direct linkage of chromatin accessibility changes to transcriptional outputs within the same cell [126].

#### 4.2.2 Unpaired multimodal profiling with computational integration

Unpaired approaches profile different molecular layers from separate cell populations generated in independent experiments. Computational algorithms are then used to align and integrate the resulting datasets into a joint embedding space [127]. Because this integration depends on computational alignment rather than on direct cellular pairing, specialized analytical frameworks are required to manage modality‐specific variations. Widely used tools such as Seurat, Harmony, and Multi‐Omics Factor Analysis (MOFA+) address this challenge by applying distinct mathematical strategies to identify shared sources of variation across modalities [128]. These frameworks serve different purposes within cross‐modal integration. Seurat is commonly applied when the primary objective is dataset alignment and cross‐modal identity transfer [129]. Harmony is particularly useful in large or heterogeneous cohorts, where controlling batch effects while preserving biological variation is essential [130]. By contrast, MOFA+ is often used to uncover coordinated patterns across omics layers, enabling the identification of latent regulatory programs otherwise inaccessible through alignment‐based approaches alone [131].

Together, these computational integration strategies have advanced our understanding of therapy‐induced epigenetic plasticity in diverse cancer types. Using scRNA‐seq and scATAC‐seq, one study identified seven epigenetically defined gene signatures in enzalutamide‐treated prostate cancer cells. These signatures distinguished drug‐responsive phenotypes from pre‐existing drug‐resistant phenotypes [109]. In addition, prognostic validation in The Cancer Genome Atlas cohort and drug–gene network analysis further suggested combinatorial strategies to overcome resistance [132]. In longitudinal neuroblastoma profiling, single‐nucleus RNA sequencing combined with a single‐nucleus assay for transposase‐accessible chromatin sequencing and spatial omics were used to profile 22 matched pre‐ and postchemotherapy patient samples. This analysis revealed a therapy‐induced expansion of adrenergic (ADRN)‐calcium and ADRN‐dopaminergic states and a reduction in proliferative states [27]. Chromatin accessibility at the MYCN locus served as a marker of clinically unfavorable epigenetic states [27]. In KMT2A‐rearranged leukemia, computational integration of unpaired scRNA‐seq and scATAC‐seq datasets delineated increased epigenetic plasticity and the presence of stem‐like blast populations. These stem‐like states were preferentially associated with therapy‐resistant phenotypes, highlighting the utility of multi‐omics integration for capturing clinically relevant adaptive trajectories [133]. In metastatic melanoma, cellular indexing of transcriptomes and epitopes by sequencing (CITE‐seq) was combined with matched 40‐plex PhenoCycler tissue imaging for multimodal profiling. This approach identified an immune‐striving tumor microenvironment with epigenetic signatures associated with innate and acquired immunotherapy resistance [134]. The principal strength of unpaired approaches is their broad applicability and flexibility, enabling comparative analyses of pre‐ and post‐treatment epigenetic landscapes across large patient cohorts. Because unpaired integration captures molecular layers from different cells, it depends on computational assumptions. This can make it difficult to accurately resolve rare or transient cell populations associated with the development of therapeutic resistance.

### 4.3 Technical considerations and limitations in comparative frameworks

Single‐cell platforms and integration strategies entail technical trade‐offs that critically influence the study design and data interpretation in the context of therapy‐induced epigenetic plasticity profiling (Table 2). Paired approaches offer superior resolution for mechanistic studies requiring direct epigenetic–transcriptional coupling but are associated with lower cell throughput, higher technical complexity, and are currently limited to measuring mainly two modalities simultaneously [120, 121, 127]. Unpaired computational approaches enable the analysis of larger sample sizes and multi‐condition comparisons. However, they rely on computational alignment across different cells. This process can obscure subtle distinctions and hinder the detection of rare or transitional cell states [135]. In addition to integration strategies, individual platforms have distinct limitations. The scATAC‐seq data are sparse at the single‐cell level and often require pseudo‐bulk aggregation for reliable peak calling. This reduces sensitivity to subtle chromatin accessibility changes in low‐abundance cell populations [136]. scCUT&Tag and scChIP‐seq remain technically demanding at low cell numbers and may exhibit antibody‐dependent background signals that complicate histone modification quantification [110]. Single‐cell methylome sequencing provides the most direct measure of DNA methylation but suffers from low CpG coverage per cell, often requiring imputation strategies that limit the resolution and accuracy of methylation inference at sparsely covered loci [111, 137]. A key challenge in studying therapy‐induced plasticity is the difficulty of detecting transient or intermediate cell states, which may appear only temporarily during epigenetic reprogramming and can be overlooked in cross‐sectional sampling approaches. Single‐cell multi‐omic analysis of CLL relapse during therapy with venetoclax, a BCL2 inhibitor, demonstrated coordinated epigenetic and transcriptional adaptations associated with resistance. These adaptations were detectable only through simultaneous multimodal profiling, highlighting the importance of selecting appropriate platforms based on the biological question [138]. Different platforms are suitable for detecting rare adaptive states, mapping regulatory element activity, and characterizing the repressive chromatin configurations associated with resistance, depending on the underlying biological question.

**Table 2:** Comparison of single‐cell multi‐omics platforms for epigenetic profiling.

Example method	Molecular layers profiled	Key strength	Major limitations	Relevance to epigenetic plasticity	References
scATAC‐seq	Chromatin accessibility	Identifies regulatory elements and TF motif activity	Sparse signal, limited transcript linkage	Maps enhancer remodeling during therapy	[136]
scCUT&Tag, scChIP‐seq	Histone marks (H3K27me3, H3K4me3)	Direct profiling of chromatin state	Limited throughput	Reveals lineage plasticity and chromatin repression patterns	[110]
scRNA‐seq + scATAC‐seq	Transcriptome + chromatin accessibility	Identification of resistance‐associated epigenetic states	Indirect link to gene expression	Adaptive chromatin states in resistance	[20, 109]
10x Multiome	RNA + Chromatin accessibility	Direct regulatory linkage within same cell	Higher cost	Defines active regulatory networks in resistant states	[123]
Paired‐Tag	RNA + histone modification	Connects chromatin repression to transcription	Technical complexity	Maps epigenetic silencing in immune‐evasive states	[121]
scM&T‐seq	DNA methylation + RNA	Links methylation changes to gene expression	Low throughput	Identifies methylation‐driven therapy adaptation	[122]
Spatial ATAC + RNA	RNA + accessibility (spatial)	Maps regulatory plasticity in the microenvironment	Limited tissue coverage and resolution	Dissects therapy‐driven TME interactions	[144]
snRNA + snATAC	RNA + accessibility	Suitable for frozen clinical samples	Lower RNA sensitivity	Enables profiling of treated patient biopsies	[27]

Beyond platform‐specific constraints, several experimental and interpretational limitations should be considered when applying single‐cell multi‐omics approaches to study therapy‐induced epigenetic plasticity. First, tissue dissociation can introduce systematic biases, as vulnerable cell populations, particularly fragile or therapy‐stressed cells, may be preferentially lost during sample preparation [139]. This can lead to underrepresentation of clinically relevant transient states. Second, the use of fresh versus frozen samples introduces additional variability. Although frozen samples increase feasibility for clinical studies, they may compromise chromatin accessibility and RNA integrity, thereby affecting data quality and cross‐modal integration [139, 140]. Temporal resolution also represents a major limitation. Most single‐cell studies rely on cross‐sectional sampling, which captures static snapshots rather than dynamic trajectories of cell state transitions [72]. Consequently, transient intermediate states during therapy‐induced reprogramming may be missed or inaccurately inferred. Approaches such as longitudinal sampling and lineage tracing can partially address this limitation by enabling the reconstruction of cell state trajectories and clonal relationships over time [141, 142].

In addition, the lack of spatial context in most single‐cell multi‐omics datasets limits the ability to interpret interactions between tumor cells and the microenvironment [143]. Recent advances in spatially resolved multi‐omics technologies have provided an opportunity to link epigenetic states with tissue architecture, which is particularly relevant for understanding immune‐evasive or niche‐dependent adaptive states [144]. Finally, a fundamental challenge lies in inferring causality from integrated multi‐omics data. Although epigenetic features such as chromatin accessibility, DNA methylation, and histone modifications have been shown to correlate with gene expression, these relationships alone are insufficient to define direct regulatory mechanisms [135]. Without perturbation‐based validation, such as CRISPR‐mediated epigenetic editing or functional assays, distinguishing causal drivers from downstream consequences remains a challenge [145, 146]. Therefore, single‐cell multi‐omics should be integrated with functional perturbation frameworks to establish mechanistic links between epigenetic remodeling and therapy resistance.

## 5 From epigenetic profiling to therapeutic decision‐making

Single‐cell multi‐omics analysis has provided important insights into therapy‐induced epigenetic plasticity and its clinical implications [24, 147]. Therefore, a structured framework is required to translate these molecular insights into therapeutic strategies. This framework involves three sequential steps (Fig. 4). First, tumor populations must be profiled at single‐cell resolution to define the spectrum of cellular and epigenetic states before and after therapy. Second, state‐specific vulnerabilities must be systematically characterized by connecting distinct epigenetic configurations with their associated transcriptional outputs and metabolic dependencies. Finally, translating these multi‐omics insights into a clinical decision‐making framework represents the final and most critical step toward precision oncology.

**Fig. 4:**
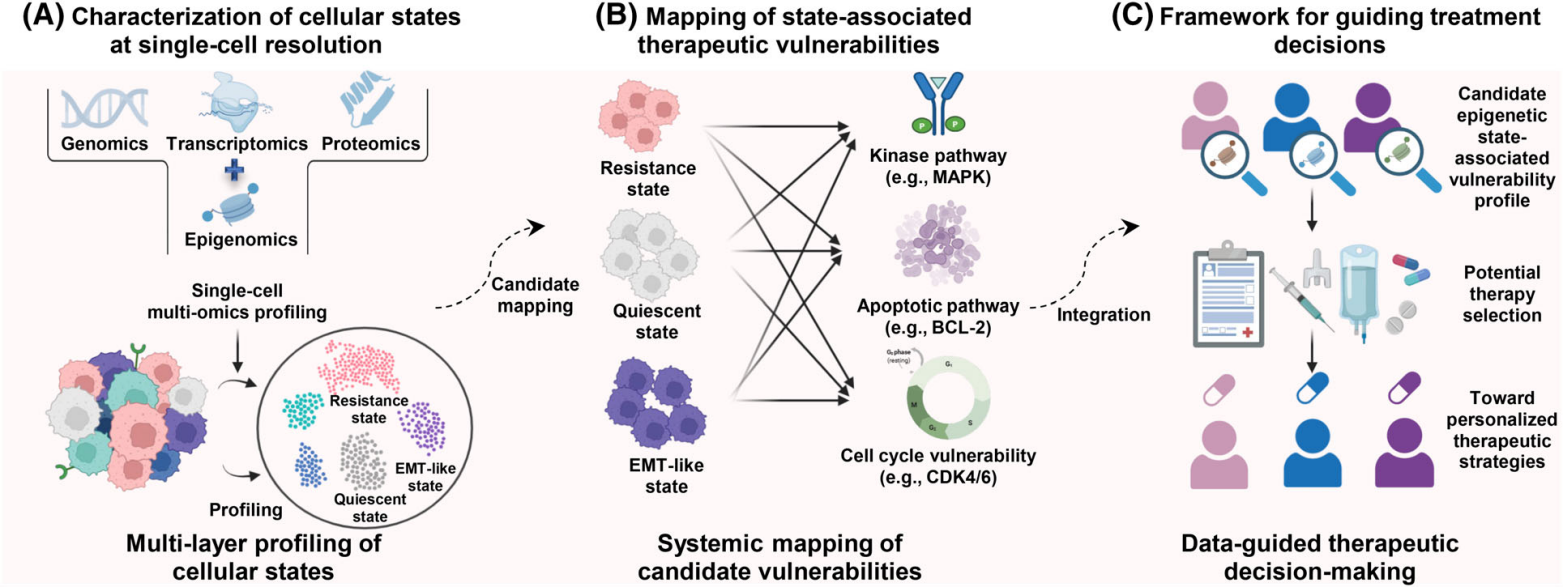
Adaptive precision oncology framework driven by single‐cell multi‐omics. Conceptual framework illustrating how single‐cell multi‐omics can inform adaptive, state‐guided therapeutic strategies in precision oncology. (A) Single‐cell multi‐omics profiling integrates epigenomic, transcriptomic, and proteomic layers to characterize heterogeneous tumor cell states present before and after therapy. Distinct adaptive phenotypes, including resistant, epithelial–mesenchymal transition (EMT)‐like, and quiescent states, can be resolved at single‐cell resolution, though considerable overlap and intermediate states are frequently observed. (B) Integration of multi‐omic features may facilitate identification of state‐associated therapeutic dependencies. Adaptive states may exhibit preferential associations with state‐dependent regulatory and metabolic vulnerabilities, including kinase signaling pathways, anti‐apoptotic dependencies, and cell cycle regulators. However, these dependencies are frequently shared across states and should be interpreted in the context of tumor heterogeneity and plasticity. (C) Multi‐omics vulnerability mapping informs data‐driven therapeutic decision‐making. Selection of targeted therapies, cytotoxic agents, or immunotherapies is guided by the dominant adaptive states and their associated dependencies. Iterative profiling enables refinement of therapeutic strategies over time. Integrated multi‐omics data serves as the foundational layer supporting this precision oncology framework.

### 5.1 Multi‐omics biomarkers for precision oncology

The first step in this framework is the identification of biomarkers that capture therapy‐induced cellular states. Single‐cell multi‐omics enables the identification of integrated epigenetic and transcriptional profiles associated with distinct adaptive phenotypes (Fig. 4A) [148, 149]. In contrast to bulk‐level molecular analyses, multi‐layer single‐cell profiling links chromatin accessibility, DNA methylation, histone modifications, and gene expression programs across defined cellular states. These integrated signatures can serve as predictive biomarkers for treatment response, relapse risk, or minimal residual disease [150, 151]. By capturing coordinated regulatory programs rather than individual genomic or epigenomic alterations, multi‐omics biomarkers offer improved specificity and functional interpretability in precision oncology [152]. Within this framework, several epigenetic features linked to adaptive cell states have been identified as promising candidate biomarkers. For instance, chromatin accessibility profiles of pre‐existing DTP‐like subpopulations have been proposed as early indicators of resistance potential, suggesting that pretreatment epigenetic heterogeneity could help stratify patients by resistance risk [108, 123]. Similarly, DNA methylation patterns associated with senescence‐like and quiescent states have been explored as indicators of residual disease burden following cytotoxic therapy [153]. In this context, the epigenetic maintenance of quiescence may represent a mechanism by which dormant tumor cells evade therapeutic elimination.

Epigenetic heterogeneity has been proposed as a measurable biomarker. In gliomas, integration of single‐cell DNA methylome and transcriptome data revealed increased methylation disorder under therapeutic stress, which was linked to transcriptional disruption and faster disease progression [154]. In addition, histone modification landscapes may provide predictive information. H3K27me3 distribution patterns at lineage‐specifying loci have been linked to cell fate determination at the onset of cytotoxic therapy [25]. In triple‐negative breast cancer, pre‐existing H3K27me3 landscapes have been shown to establish a permissive epigenetic configuration for persister cell programs prior to drug exposure, supporting their potential role as predictors of therapy resistance [25]. Polycomb‐dependent chromatin regulation has also been implicated in lineage reprogramming in hormone therapy‐resistant cancers, particularly in castration‐resistant prostate cancer, where lineage plasticity underlies therapeutic escape [155]. Integration of scRNA‐seq and chromatin accessibility profiling also supports drug repositioning strategies based on epigenetic state information. In breast cancer, this approach uncovered dysregulated immunomodulatory peptides in malignant epithelial cells and suggested candidate compounds targeting beta‐2‐microglobulin and secretory leukocyte protease inhibitors to enhance antitumor immunity. This illustrates how multi‐omics profiling can extend therapeutic opportunities by revealing resistance mechanisms that may be overlooked by conventional strategies [156]. Collectively, Polycomb‐mediated repression may function as a shared regulatory axis linking dormancy stabilization and lineage plasticity during therapy adaptation. Integrated transcriptomic and chromatin accessibility profiling at single‐cell resolution enables identification of regulatory elements and TF networks that are selectively active in persister or resistant cell populations [157]. These molecular features represent candidate biomarkers that warrant clinical validation through tumor biopsy‐based assays or, prospectively, through liquid biopsy platforms as single‐cell epigenomic technologies continue to mature. To facilitate a more structured translational interpretation, representative case studies are summarized in Table 3 and organized according to their level of evidence and proximity to clinical implementation. Multi‐omics profiling also enables systematic vulnerability mapping within distinct adaptive states, as discussed in the following section.

**Table 3:** Clinically relevant epigenetic signatures and biomarker candidates from single‐cell multi‐omics studies.

Cancer type	Multi‐omic platform	Evidence level	Identified biomarker signature	Clinical relevance	References
Pediatric B‐ALL	ATAC‐seq + scRNA‐seq + CyTOF	Clinical trial‐supported	Stem cell‐like epigenome with PRC2‐target hypermethylation and reduced chromatin accessibility	Candidate pretreatment biomarker of primary nonresponse to CD19 CAR‐T therapy	[159]
CLL	scRNA‐seq + single‐cell long‐read RNA‐seq	Patient cohort‐supported	Multilayered resistance program with recurrent NF‐κB activation and anti‐apoptotic rewiring	Candidate biomarker of venetoclax relapse and treatment adaptation	[138]
Medulloblastoma	Single‐nucleus multiome (snRNA‐seq + snATAC‐seq)	Preclinical/proof‐of‐concept	Persistent progenitor population with metabolism‐linked epigenetic reprogramming	Candidate biomarker of therapy persistence and state‐linked vulnerability to metabolic targeting	[123]
Multiple myeloma	Single‐nucleus multiome (RNA‐seq + ATAC‐seq)	Patient cohort‐supported	Epigenetic silencing of GPRC5D regulatory regions	Resistance‐associated epigenetic target inactivation under GPRC5D‐directed T‐cell engager therapy	[124]
Breast cancer	scRNA‐seq + scATAC‐seq	Patient cohort‐supported	Epigenetically defined resistant cancer cell states and heterogeneity‐guided core signature	Candidate marker for endocrine resistance and recurrence‐associated heterogeneity	[20]
Colorectal cancer	scRNA‐seq + scATAC‐seq + single‐cell DNA profiling	Preclinical/proof‐of‐concept	Heritable epigenetic resistance memory	Candidate marker of durable resistant states and relapse potential	[125]
Ovarian cancer	snRNA‐seq + snATAC‐seq	Patient cohort‐supported	Open chromatin signature in persister tumor cells	Candidate epigenetic signature of drug‐tolerant persister cells	[157]
Prostate cancer	scRNA‐seq + scATAC‐seq	Patient cohort‐supported	Pre‐existing and persistent chromatin‐defined cell states linked to lineage plasticity	Candidate predictor of relapse and resistance to AR‐targeted therapy	[109]

Evaluating the translational potential of these findings requires attention to both the level of supporting evidence and the clinical readiness of each biomarker category [158]. In this context, representative single‐cell multi‐omics signatures and biomarker candidates can be broadly classified into three groups: clinical trial‐supported biomarkers, patient cohort‐supported biomarkers, and preclinical or proof‐of‐concept candidates [159]. Clinical trial‐supported biomarkers have been evaluated in prospective or trial‐associated settings; however, these examples remain rare in the context of single‐cell multi‐omics. Patient cohort‐supported biomarkers have been associated with treatment response, relapse, and resistant cell states in clinical samples. However, further validation is required before routine clinical use [20, 109]. Preclinical or proof‐of‐concept candidates are primarily derived from experimental models and provide mechanistic insights into adaptive cell states while remaining far from clinical implementation [157]. To provide a more critical translational perspective, representative examples are categorized in Table 3 according to this framework. This classification highlights that, although many multi‐omic signatures provide substantial biological insight, only a limited subset currently approaches clinical translation, underscoring the need for systematic validation, assay standardization, and clinically adaptable testing strategies [158].

### 5.2 Rational design of strategies targeting epigenetic plasticity

The utility of single‐cell multi‐omics extends to the systematic mapping of state‐associated therapeutic vulnerabilities, providing a rational framework for designing therapeutic strategies that limit adaptive‐state transitions (Fig. 4B). In addition, dependency mapping based on integrated single‐cell multi‐omics data can reveal molecular targets that are essential for adaptive cell states. These state‐specific requirements are often masked in bulk tumor analyses, where signals from resistant subclones are diluted by the dominant cell population [160]. Therefore, single‐cell approaches play a central role in the precise identification of adaptive‐state‐specific therapeutic targets.

Several vulnerability‐mapping strategies have been developed through single‐cell epigenetic studies. EZH2 inhibition has been proposed as a selective vulnerability in lineage‐reprogrammed, H3K27me3‐enriched resistant states, because these cells rely on sustained PRC2 activity to maintain repression of lineage‐specific programs [161]. BET bromodomain inhibitors targeting BRD4‐driven enhancer activation represent potential therapeutic strategies for cells that exhibit adaptive activation of stress‐responsive enhancers [162]. In EMT‐associated resistance states, reliance on the SWI/SNF complex has been identified through integrated chromatin accessibility and transcriptional profiling. This analysis revealed targetable dependencies on SMARCA4/BRG1 and SMARCA2 [29]. TET enzyme‐mediated demethylation pathways have been implicated in quiescent and DTP‐like states, suggesting that disruption of DNA methylation homeostasis may affect maintenance of these epigenetic programs [163]. Sequential or combined targeting of shared epigenetic mechanisms has shown preclinical efficacy in reversing adaptive resistance. For example, combining DNMT and HDAC inhibitors disrupts DNA methylation‐ and histone modification‐based repressive states [164]. Multi‐omics profiling can help define how epigenetic and transcriptional states shift in response to therapy, thereby informing adaptive or sequential treatment strategies aimed at limiting the development of secondary resistant states [27].

Similar to biomarker development, the translational potential of state‐specific vulnerabilities depends on the level of supporting evidence and the feasibility of therapeutic targeting. Many vulnerabilities identified through single‐cell multi‐omics analyses, such as dependencies on PRC2 activity, enhancer regulation, and chromatin remodeling complexes, are supported primarily by preclinical studies and functional perturbation experiments [157, 161]. These findings provide important mechanistic insights but often lack direct clinical validation. A smaller subset of these targets is linked to pathways for which clinically approved or investigational inhibitors already exist, including epigenetic regulators such as DNMTs, HDACs, and BET proteins [165]. In these cases, multi‐omics data can inform patient stratification or combination strategies, thereby enhancing the translational relevance of these targets. Accordingly, the translational relevance of these vulnerabilities should be interpreted in the context of their level of experimental support, pharmacological tractability, tissue accessibility, and the feasibility of incorporation into clinically realistic treatment strategies. However, the context‐dependent nature of epigenetic plasticity presents a challenge, as the same target may have different effects depending on tumor type, treatment history, and cell state composition [166]. Therefore, bridging the gap between vulnerability identification and clinical implementation requires integration of multi‐omics profiling with functional validation, pharmacological feasibility, and carefully designed clinical trials.

### 5.3 Clinical implementation challenges

Single‐cell multi‐omics–guided therapeutic decision‐making holds considerable promise within an adaptive precision oncology framework, but further progress is needed for broader clinical use (Fig. 4C). Practical barriers at multiple stages hinder clinical implementation. Another major barrier is assay readiness. Many candidate biomarkers identified through single‐cell multi‐omics studies still depend on complex research‐grade workflows that remain technically demanding for routine clinical testing.

Technical challenges include the requirement for fresh or minimally processed tissue to achieve high‐quality single‐cell profiling, limited applicability to surgically accessible tumors, and complicated longitudinal monitoring. The high cost and computational demands of multi‐omics data generation and integration remain prohibitive for clinical deployment. In addition, the absence of standardized analytical pipelines across institutions introduces variability that undermines the reproducibility of biomarker signatures [167]. Clinical validation of epigenetic biomarkers identified in preclinical single‐cell studies requires prospective cohort studies with sufficient sample sizes to establish analytical validity and clinical utility across diverse patient populations and cancer types [168].

However, biological complexity presents additional challenges. Therapy‐induced epigenetic states are highly dynamic and context‐dependent. The same molecular signature can have different prognostic implications depending on tumor type, treatment history, and microenvironmental context. The reversibility of epigenetic states offers therapeutic opportunities, while complicating biomarker interpretation. This is because biopsy samples capture only a snapshot of the epigenetic landscape at a given time and may provide an incomplete view of the evolving plasticity trajectory in tumors.

Several technological and analytical advances are poised to accelerate clinical translation. The development of minimally invasive epigenomic profiling approaches, including cell‐free DNA methylation sequencing and single‐cell analysis of circulating tumor cells, may enable longitudinal monitoring of therapy‐induced epigenetic state transitions without repeated tissue biopsies [169]. Integration of spatial multi‐omics with single‐cell profiling will provide additional resolution of the microenvironmental context shaping epigenetic plasticity, while the application of machine learning frameworks to multi‐omics datasets holds promise for identifying composite biomarker signatures with superior predictive performance. The clinical translation of single‐cell multi‐omics for precision oncology requires coordinated efforts across technology development, computational biology, and clinical trial design. These efforts are essential to establish the framework for clinical adoption.

## 6 Conclusions and future perspectives

Single‐cell multi‐omics technologies provide an unprecedented resolution of epigenetic plasticity and its role in therapeutic adaptation. By integrating transcriptional and epigenetic information at single‐cell resolution, these approaches illuminate the dynamic regulatory mechanisms underlying therapy‐induced tumor plasticity. Despite their conceptual power, important limitations remain in distinguishing correlations from causation within multilayered datasets. Many observed epigenetic–transcriptional associations have been inferred computationally rather than experimentally, underscoring the need for functional perturbation studies to confirm mechanistic dependencies.

However, significant technical, financial, and analytical challenges have limited their widespread clinical implementation. Variability in tissue processing, sequencing depth, and computational pipelines can substantially influence inferred cellular states, raising concerns regarding cross‐cohort reproducibility and standardization. Overcoming these barriers will require the standardization of analytical frameworks, development of minimally invasive profiling strategies, and rigorous prospective validation of multi‐omics biomarkers. Furthermore, the scalability of high‐dimensional multi‐omics profiling in routine oncology practice needs to be established, particularly in resource‐limited clinical settings. The integration of complex datasets into interpretable decision‐support tools for clinicians presents an additional translational hurdle.

As technologies mature and paired multimodal approaches become more accessible, direct characterization of epigenetic–transcriptional coupling will become increasingly feasible. This will help to identify therapeutic vulnerabilities and guide more effective treatment strategies. The reversibility of epigenetic states, which complicates biomarker interpretation, offers a unique therapeutic opportunity. Simultaneously, the dynamic and context‐dependent nature of epigenetic plasticity suggests that static profiling may be insufficient to fully capture resistance trajectories, emphasizing the importance of longitudinal sampling and adaptive clinical trial designs. Ultimately, integrating single‐cell multi‐omics into clinical trial designs and precision oncology workflows may enable more adaptive and durable strategies for overcoming treatment resistance. Clinical progress depends on coordinated advances in technological development, computational modeling, experimental validation, and prospective clinical evaluation. Together, these advances will help translate epigenetic insights into meaningful clinical applications.

## Conflict of interest

The authors declare no conflict of interest.

## 7 Author contributions

HJK and DP were involved in conceptualization. HJK, HL, JH, and DP were involved in writing of the manuscript and literature review. All authors reviewed the manuscript.
